# Editorial: Effects of Abnormal Metabolism on Germ Cell Growth and Maturation, and on Fertilization in Mammals

**DOI:** 10.3389/fvets.2022.962334

**Published:** 2022-07-06

**Authors:** Yi Fang, Xiangwei Fu, Qien Yang, Shoulong Deng, Xianlong Wang

**Affiliations:** ^1^Jilin Provincial Key Laboratory of Grassland Farming, Northeast Institute of Geography and Agoecology, Chinese Academy of Sciences, Changchun, China; ^2^China Agricultural University, Beijing, China; ^3^Key Laboratory of Adaptation and Evolution of Plateau Biota, Northwest Institute of Plateau Biology, Chinese Academy of Sciences, Xining, China; ^4^NHC Key Laboratory of Human Disease Comparative Medicine, Peking Union Medical College, Institute of Laboratory Animal Sciences, Chinese Academy of Medical Sciences and Comparative Medicine Center, Beijing, China; ^5^Baylor College of Medicine, Houston, TX, United States

**Keywords:** metabolism, sperm, oocyte, infertility, gene expression

Dynamic regulation of metabolism is critical for germ cell development and fertilization. Each process and key event in germ cell maturation and fertilization potentially requires a unique substrate and metabolic pathway, depending on the species. These pathways are particularly sensitive to changes in nutritional, chemical and endocrine environments, as well as metabolite concentrations and enzyme activities. Specifically, decreased concentrations of transport proteins and increases in glucose/lipid contents and reactive oxygen species have been implicated in meiotic defects, mitochondrial dysfunction, and epigenetic alterations, affecting germ cell maturation and development. Therefore, this special issue focuses on research highlights and challenges in metabolic events affecting mammalian germ cell growth, protection, and preservation, as well as reproductive diseases, etc. Furthermore, it also provides new insights regarding treatment of infertility caused by metabolic diseases.

It is well established that sustaining metabolism of post-thaw sperm is important for acceptable fertility following AI. High (153 mM) glucose concentrations during cooling promoted glycolysis and increased lactate concentrations in boar sperm, and resulted in sperm with circular movements. In contrast, reduced glucose concentrations suppressed sperm glycolysis and increased the activity of key enzymes in oxidative phosphorylation, as well as increased ATP concentrations. Based on those observations, a low glucose concentration (30.6 mM) was deemed suitable for cryopreservation of boar semen (Zhu et al.).

Metabolic dysfunction and obstacles to communications between an oocyte and its surroundings due to aging, heat stress (HS), disease, etc., are associated with oocyte defects and infertility. Subclinical endometritis reduced oocyte nuclear maturation and fertilization in repeat breeder (RB) cows and inhibited expressions of GDF9, StAR and FSHr, resulting in a poor microenvironment for the final stages of oocyte development in pre-ovulatory follicles of RB cows (Kafi et al.). Testicular HS can influence testicular concentrations of amino acids, fatty acids, minerals, and antioxidants and enzyme activities. Turpan black sheep had substantial resistance to HS, whereas Suffolk sheep were much more susceptible to HS, with acrosome damage, a high proportion of sperm with DNA fragmentation, and many spermatogenic cells blocked in the zygotene and pachytene stages, followed by increased apoptosis. The PI3K-Akt-mTOR pathway is a main signaling pathway regulating spermatogonia proliferation, protein synthesis and energy metabolism by regulating expression of p70S6K and 4EBP1 in testis (Song et al.). In mice, cortical tension-related proteins pERM and pMRLC were aberrantly expressed in aged oocytes, causing decreased cortical tension. However, 5 μg/mL procyanidins (PCB2) antagonized aging-induced decreased cortical tension and protected oocytes. In addition, abnormal spindle formation and chromosome arrangement induced by cortical tension and oxidative stress in aged oocytes were also corrected by PCB2. By improving viability in vitrified-thawed oocytes, PCB2 may contribute to maintain mitochondrial function and ATP concentrations in aged oocytes (Zhuan et al.).

Endometriosis (EMs), an estrogen-dependent disease, is characterized by the appearance of the endometrium outside the uterus. The Meorangered1 module was most significantly related to infertile women with EMs. Whereas 40% of the pathways involve metabolism, intersection genes were mostly enriched in various amino acids and in the cGMP-PKG and cAMP signaling pathway. In addition, 13 miRNAs and 2 lncRNAs linked to infertility were identified to create a ceRNA regulatory network linked to infertile EMs. A strong correlation between nutrition and reproduction indicated that dietary amino acid intake influenced key molecules involved in a range of biological processes during conception (Li et al.). Therefore, clarifying associations between EMs and recurrent pregnancy loss (RPL) is a key to solving infertility, as abnormal energy metabolism may be a common pathogenic mechanism. RAB8B, GNAQ, H2AFZ, SUGT1 and LEO1 could be therapeutic candidates for RPL and EMs. The PI3K-Akt signaling pathway and platelet activation were potentially involved in EMs-induced RPL (Ye et al.).

Large-scale nuclear and cytoplasmic reorganizations require a massive amount of energy, potentially derived from a variety of substrates such as carbohydrates, amino acids, and lipids. Since these physiological processes are inherently multicellular and multi-stage, regulatory mechanisms, especially in germ cells at special stages of development, are still largely unknown. Through the proteomic profiles of porcine immature and *in vitro* mature oocytes, 237 of 763 differential proteins were classified in “metabolic process,” and assigned to the following: “energy production and conversion;” “carbohydrate transport and metabolism;” “amino acid transport and metabolism;” and “lipid transport and metabolism,” etc. Many proteins (e.g., methylsterol monooxygenase 1 and phosphatidate phosphatase) were implicated in in the signaling pathway of lipid metabolism during oocyte maturation (Jia et al.). Spermiogenesis is regulated by nutrients or cellular metabolism. Sugp2, selected as a spermatogenesis regulation-related protein, was enriched in the nucleus of male germ cells, acted as a chromatin-associated protein, and participated in regulation of complexed alternative splicing during gametogenesis. However, Sugp2 had limited effects on meiotic progression and fertility (Zhan et al.). There are indications that microRNA has important roles in male reproductive organ function. Changes in miRNA and mRNA profiles in the testes of juvenile, adolescent, adult, and aged sika deer were evaluated. Differentially expressed mRNA (IGF1R, ALKBH5, Piwil, etc) and miRNA (miR-140, miR-145, etc,) had important roles in regulating proliferation of spermatogonia and glycolysis through MAPK, p53, PI3K-Akt, and Hippo signaling pathways, etc. Furthermore, glycerophospholipid metabolism was only overlapping pathway throughout the life cycle. In addition, miR-140 was confirmed to directly target mutant IGF1R-3'UTR by Luciferase reporter assays (Jia et al.).

In summary, the above-mentioned studies collectively represent an enormous amount of new data on metabolic mechanisms of spermatogenesis, sperm cryopreservation, oocyte aging, and endometriosis in various species. Although this e-book cannot account for all metabolic disorders and characteristics of germ cells, they provide valuable information for understanding these special cells. In the context of the current focus on molecular mechanisms of key biological processes or events at gene and protein levels, it is hoped that this e-book will broaden research perspectives, and try to identify metabolism-based solutions for infertility.

## Author Contributions

YF wrote the Introduction and the Conclusion. XF, SD, QY, and XW wrote the central part with comments to the cited papers. All authors contributed to the article and approved the submitted version.

## Funding

This topic was supported by the Strategic Priority Research Program of the Chinese Academy of Sciences (XDA27040302).

## Conflict of Interest

The authors declare that the research was conducted in the absence of any commercial or financial relationships that could be construed as a potential conflict of interest.

## Publisher's Note

All claims expressed in this article are solely those of the authors and do not necessarily represent those of their affiliated organizations, or those of the publisher, the editors and the reviewers. Any product that may be evaluated in this article, or claim that may be made by its manufacturer, is not guaranteed or endorsed by the publisher.

